# Proactive identification of rare diseases: lessons from hereditary angioedema diagnosis using electronic medical records

**DOI:** 10.1186/s13023-025-03882-2

**Published:** 2025-07-28

**Authors:** Xue Wang, Huizhen Jiang, Ziyang Huang, Chao Dong, Weiguo Zhu, Shuyang Zhang, Yuxiang Zhi

**Affiliations:** 1 Department of Allergy & Clinical Immunology, Peking Union Medical College, Peking Union Medical College Hospital, Chinese Academy of Medical Sciences, National Clinical Research Center for Immunologic Diseases, Beijing, China; 2 Department of Information Center, Peking Union Medical College, Peking Union Medical College Hospital, Chinese Academy of Medical Sciences, Beijing, China; 3 Department of Primary Care and Family Medicine, State Key Laboratory of Complex Severe and Rare Diseases, Peking Union Medical College Hospital, Chinese Academy of Medical Sciences and Peking Union Medical College, Beijing, China; 4 Department of Cardiology, Peking Union Medical College Hospital, Chinese Academy of Medical Sciences and Peking Union Medical College, Beijing, China

**Keywords:** Proactive diagnosis, Electronic medical records, Hereditary angioedema, Rare diseases

## Abstract

**Background:**

Diagnosing rare diseases traditionally requires patients to endure lengthy and challenging journeys to find specialists familiar with their conditions. This study advocates a paradigm shift in rare disease diagnosis, moving from patients seeking physicians to physicians actively identifying patients. Using hereditary angioedema (HAE) as an example, we demonstrate how this approach, supported by electronic medical records (EMRs), enables proactive care for patients with rare diseases. Our EMR system incorporates a free-text search engine to screen for patients with potential HAE based on clinical symptoms and laboratory tests. Search terms include recurrent skin edema, abdominal pain, laryngeal edema, and/or decreased C4 levels. Suspected cases are followed up by telephone calls from trained physicians, inviting patients to undergo confirmatory C1-INH and C4 testing and genetic testing to ensure accurate diagnosis and appropriate treatment.

**Results:**

Of 2,689 patients who met the screening criteria, 3,441 records were analyzed. Ninety-five patients had already been diagnosed with HAE. After excluding those with a known etiology for edema or characteristics inconsistent with HAE, three patients with unexplained cutaneous edema, abdominal pain, and/or laryngeal edema were included in the final screening. Laboratory tests confirmed HAE in all three, highlighting the effectiveness of this proactive approach.

**Conclusions:**

This study underscores the transformative potential of EMRs in diagnosing rare diseases. By shifting the responsibility of identifying rare diseases from patients to healthcare professionals, we expedite diagnosis and exemplify the spirit of service in medicine, ensuring patients with rare diseases receive timely and effective care.

## Highlights

1. What is already known about this topic?

Traditional rare disease diagnosis is prolonged and complex, often requiring multiple consultations and leading to misdiagnoses. EMRs can improve diagnosis by identifying patterns in clinical data.

2. What does this article add to our knowledge?

This article demonstrates how EMRs can be used proactively to identify and diagnose rare diseases such as HAE, reducing diagnostic time through the initiative of healthcare professionals.

3. How does this study impact current management guidelines?

This study introduces a paradigm shift in diagnostic workflows for rare diseases, reducing diagnostic delays and ensuring that patients receive timely and appropriate treatment.

## Background

Rare diseases, also known as orphan diseases, are conditions that are often debilitating and life-threatening [1]. The World Health Organization defines rare diseases as those affecting fewer than 6.5 to 10 per 10,000 people [2]. To date, more than 10,000 rare diseases have been identified [3]. Patients with rare diseases constitute as much as 5.9% of the global population [4]. Increasingly, there is significant social pressure to improve diagnostic methods for rare diseases.

The diagnosis of rare diseases is challenging because of their extremely low prevalence. Most primary care physicians have likely never encountered a patient with such conditions [5]. Additionally, some rare diseases present with clinical features resembling common diseases, often leading to frequent misdiagnoses and delayed diagnoses for these patients [5, 6]. Patients frequently visit multiple departments, undergo numerous tests, and may even receive inappropriate treatments before achieving a correct diagnosis [7]. This process not only exacerbates the suffering caused by the disease but also imposes significant financial burdens. Therefore, there is an urgent need to improve the diagnostic process for rare diseases and promote early diagnosis.

With the increasing adoption of electronic medical records (EMRs), the reuse of clinical data is creating new opportunities for diagnosing and managing patients with rare diseases [8]. The review of EMRs by specialists can assist in identifying patients with rare diseases that may have been previously misdiagnosed or undiagnosed. In this study, we utilized structured EMRs and free-text search engines to identify patients with hereditary angioedema (HAE) by repurposing data stored in the EMRs.

HAE (OMIM #106100) is a rare autosomal dominant disease [9] with an estimated prevalence of 1:50,000 in the general population, although reported ranges vary from 1:10,000 to 1:150,000 [10]. The most common cause of HAE involves either a deficiency (HAE type 1, HAE-1) or dysfunction (HAE type 2, HAE-2) of C1 inhibitor (C1-INH), leading to overproduction of bradykinin, increased vascular permeability, and eventually angioedema [9]. In addition, although very rare, HAE with normal C1-INH is now recognized and genetically identifiable [11]. Currently, only patients with HAE-1 or HAE-2 have been identified in China; no cases of HAE with normal C1-INH have been reported. The characteristic symptom of HAE is recurrent episodes of swelling that often affect the hands, feet, extremities, face, gastrointestinal tract, or upper airway [12]. Edema involving the gastrointestinal tract can cause sudden severe abdominal pain, while laryngeal edema can lead to asphyxiation [13]. Because of its rarity and the similarity of its symptoms to common diseases such as irritable bowel syndrome, small bowel obstruction, pancreatitis, or appendicitis, HAE is often underdiagnosed or misdiagnosed [14]. In one study of Chinese patients, the average delay from first attack to the diagnosis of HAE was 12.64 years, ranging from 1 month to 45 years [15]. Such delays prevent timely treatment and increase the disease burden, which can have serious consequences. Mortality from asphyxia is higher in patients with undiagnosed than diagnosed HAE [16]. A retrospective study of 728 patients with HAE from 182 families showed that 70 of 214 deaths were due to laryngeal edema, with 90% of these cases undiagnosed during the patients’ lifetimes [16]. These findings underscore the importance of early diagnosis to reduce the risk of death in patients with HAE.

Based on the urgent need to advance the diagnosis of rare diseases, our study aims to reduce the need for individuals with rare diseases to be referred to multiple specialties. We sought to enhance physician involvement by proactively utilizing their diagnostic expertise. This study uses HAE as a case study to facilitate a paradigm shift in diagnostic models for rare diseases. This initiative aims to promote early diagnosis and treatment in a way that upholds the ethos of medical care.

## Methods

### Study design

This was a single-center, retrospective analysis. An EMR system was designed and implemented with an integrated free-text search engine. By entering search terms, we can filter patients who meet specific criteria, including clinical symptoms and laboratory test results, enabling accurate and efficient screening. This study was approved by the Research Ethical Committee of the Peking Union Medical College Hospital (Number: K3640).

### Inclusion and exclusion criteria

This study aimed to identify individuals with potential HAE who presented with unexplained recurrent edema, abdominal pain, laryngeal edema, and/or reduced C4 levels. To achieve this, multiple sets of search terms were developed, and individuals identified in the EMR system through these terms were included. The specific screening criteria and search terms are detailed in Table 1. Because the EMR system was fully adopted at our hospital in 2019, the screening period was limited to January 2019 to March 2022.

**Table 1:** Search terms and detailed characteristics of patients in each group

	Search terms	Number of patients / Number of case records	Age, mean ± SD	Female, *n* (%)	Medical department (%)	Diseases (%)	Newly diagnosed HAE patients
1	Outpatients: The current medical history includes “edema”, or “puffiness”, or “swelling“ and laboratory tests include complement C4 < 0.1.	570/633	38.4 ± 16.8	451 (79.12)	Rheumatology and Immunology, 279 (48.9); Allergy, 105 (18.4); Nephrology, 86, (15.1); Internal medicine, 29 (5.1); Others, 71 (12.5)	SLE, 258 (45.3); HAE, 81 (14.2); CTD, 47 (8.2); Nephrotic syndrome, 18 (3.2); Angioedema, 15 (2.6); Others, 151 (26.5)	Patient 3
2	Outpatients: The current medical history includes “abdominal pain”, or “abdominal discomfort”, or “stomach pain”, or “abdominal cramps”, and laboratory tests include complement C4 < 0.1.	159/171	36.4 ± 15.8	124 (72.5)	Rheumatology and Immunology, 60 (37.7); Allergy, 58 (36.5); Gastroenterology, 8 (5.0); Nephrology, 7 (4.4); Emergency, 7 (4.4); Others, 19 (12)	SLE, 50 (31.4); HAE, 49 (30.8); IgG4-RD, 7 (4.4); CTD, 5 (3.1); Anaphylactic purpura, 4 (2.5); Others, 44 (27.8)	Patient 2
3	Outpatients: The current medical history includes “recurrent edema”, or “recurrent puffiness”, or “recurrent swelling“ and laboratory tests include complement C4 < 0.1.	5/5	44.8 ± 19.6	3 (60)	Allergy, 2 (40); Rheumatology and Immunology, 1 (20); Nephrology, 1 (20); Pediatrics, 1 (20)	SLE, 3 (60); HAE, 2 (40)	-
4	Outpatients: The current medical history includes “recurrent abdominal pain”, or “recurrent abdominal discomfort”, or “recurrent stomach pain”,or “recurrent abdominal cramps”, and laboratory tests include complement C4 < 0.1.	6/6	40.8 ± 13.7	5 (83.3)	Allergy, 3 (50); Rheumatology and Immunology, 2 (33.3); Chinese medicine, 1 (16.7)	HAE, 3 (50); SLE, 3 (50)	-
5	Outpatients: The current medical history includes “edema”, or “puffiness”, or “swelling“, and “abdominal pain”, or “abdominal discomfort”, or “stomach pain”, or “abdominal cramps”.	194/219	44.5 ± 20.1	136 (62.1)	Allergy, 57 (29.4); Gastroenterology, 37 (19.1); Rheumatology and Immunology, 13 (6.7); Emergency, 13 (6.7); General surgery, 13 (6.7); Others, 61 (31.4)	Anaphylaxis, 39 (20.1); Pancreatic cancer/tumor, 8 (4.1); HAE, 5 (2.6); IgG4-RD, 5 (2.6); Food allergy, 5 (2.6); Others, 132 (68)	-
6	Outpatients: The current medical history includes “edema”, or “puffiness”, or “swelling“, and “abdominal pain”, or “abdominal discomfort”, or “stomach pain”, or “abdominal cramps”, and laboratory tests include complement C4 < 0.1.	5/5	30.2 ± 18.3	1 (20)	Allergy, 2 (40); Rheumatology and Immunology, 1 (20); Emergency, 1 (20); Infectious diseases, 1 (20)	HAE, 2 (40); SLE, 1 (20); Abdominal infection, 1 (20); Cirrhosis, 1 (20)	-
7	Outpatients: Diagnosis includes “epiglottitis” or “acute epiglottitis” or “pharyngitis” or “acute pharyngitis” or “laryngeal edema”, and laboratory tests include complement C4 < 0.1.	5/5	39.4 ± 15.6	5 (100)	Allergy, 3 (60); Rheumatology and Immunology, 1 (20); ENT, 1 (20)	HAE, 3 (60); SLE, 1 (20); Acute epiglottitis, 1 (20)	-
8	Inpatients: The current medical history includes “edema”, or “puffiness”, or “swelling“ and laboratory tests include complement C4 < 0.1.	856/923	39.3 ± 18.3	670 (78.3)	Rheumatology and Immunology, 324 (37.9); Nephrology, 167 (19.5); Internal medicine, 117 (13.7); Pediatrics, 60 (7); Emergency, 25 (2.9); Others, 163 (19)	SLE, 536 (62.6); Overlap syndrome, 12 (1.4); Cryoglobulinemia, 10 (1.2); Nephrotic syndrome, 10 (1.2); IgG4-RD, 9 (1.1); Others, 279 (32.5)	Patient 1
9	Inpatients: The current medical history includes “abdominal pain”, or “abdominal discomfort”, or “stomach pain”, or “abdominal cramps”, and laboratory tests include complement C4 < 0.1.	415/453	39.6 ± 19.0	284 (68.4)	Rheumatology and Immunology, 124 (29.9); Gastroenterology, 63 (15.2); Internal medicine, 45 (10.8); Emergency, 45 (10.8); Pediatrics, 29 (7); Others, 109 (26.3)	SLE, 286 (68.9); Pancreatitis, 24 (5.8); Intestinal obstruction, 21 (5.1); Behcet disease, 12 (2.9); IgG4-RD, 11(2.7); Others, 61 (14.6)	Patient 1
10	Inpatients: The current medical history includes “edema”, or “puffiness”, or “swelling“, and “abdominal pain”, or “abdominal discomfort”, or “stomach pain”, or “abdominal cramps”, and laboratory tests include complement C4 < 0.1.	68/74	36.6 ± 14.9	53 (77.9)	Rheumatology and Immunology, 25 (36.8); Internal medicine, 12 (17.6); Gastroenterology, 8 (11.8); Nephrology, 7 (10.3); Emergency, 3 (4.4); Others, 13 (19.1)	SLE, 42 (61.8); Overlap syndrome, 2 (2.9); Cryoglobulinemia, 2 (2.9); HAE, 1 (1.5); Intestinal obstruction, 1 (1.5); Others, 20 (29.4)	Patient 1
11	Outpatients and Inpatients: Diagnosis includes “tracheotomy”, or current history includes “tracheotomy” or “laryngeal edema”, or past history includes “tracheotomy”.	180/385	54.3 ± 16.8	42 (39.3)	ENT, 153 (37.7); Emergency, 75 (18.5); Respiratory, 17 (4.2); Rheumatology and Immunology, 5 (1.2); Internal medicine, 5 (1.2); Others, 151 (37.2)	Tracheotomy, 233 (57.4); Throat cancer, 29 (7.1); Pulmonary infection, 14 (3.4); Thyroid cancer, 14 (3.4); Trauma, 9 (2.2); Others, 107 (26.5)	-

Abbreviations: ENT: ear nose and throat, SLE: systemic lupus erythematosus, HAE: hereditary angioedema, CTD: connective tissue disease, IgG4-RD: IgG4-related disease

The EMRs were independently reviewed by two evaluators. Individuals with known underlying conditions that could cause skin edema, abdominal pain, laryngeal edema, and/or reduced C4 levels were considered to have a clear etiology and were excluded from the study. These underlying conditions included rheumatic and autoimmune diseases, infectious diseases, gastrointestinal diseases, otolaryngological disorders, hematological diseases, and malignancies [14].

Individuals with recurrent skin edema, abdominal pain, laryngeal edema, and/or reduced C4 levels without a clear etiology were followed up by trained physicians via telephone. A series of questions were posed according to the international WAO/EAACI guidelines:

Question 1: Have you had a history of recurrent skin swelling without urticaria?

Question 2: Have you had a history of recurrent abdominal pain?

Question 3: Have you had a history of laryngeal edema?

Question 4: Did the symptoms appear in childhood or adolescence?

Question 5: Has treatment with antihistamines or hormones been ineffective?

Question 6: Do any of your blood relatives have similar symptoms?

If at least one of the answers to Questions 1 through 3 was “yes” and any of the answers to Questions 4 through 6 was “yes,” the individual was suspected of having HAE and was asked to undergo repeat testing for C1-INH and C4 levels. The diagnosis of HAE was confirmed based on the typical clinical presentation (recurrent skin swelling, gastrointestinal attacks, and/or laryngeal edema) and repeated laboratory tests showing abnormal C1-INH and C4 levels [17].

## Results

**Fig. 1:**
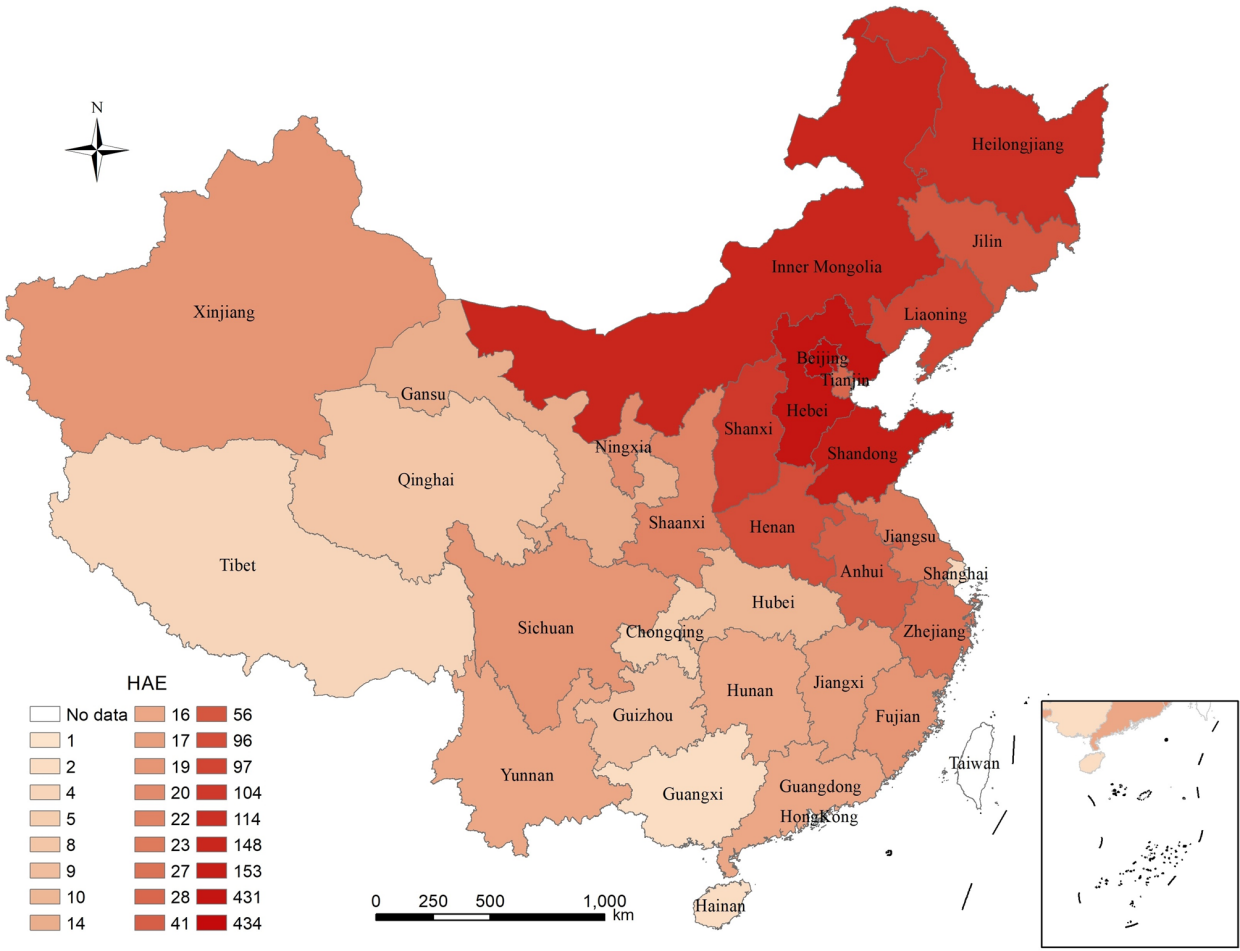
Distribution of sources for patients meeting the inclusion criteria. This map was generated using the ESRI ArcMap 10.6 software (http//:www.esri.com)

Eleven groups with different screening criteria were established. In total, 2,463 patients meeting the criteria were included in the study, providing 2,879 records. After de-duplication, 2,421 patients from various provinces of China remained (Fig. 1). Detailed characteristics of the patients in each group are shown in Table 1. Two authors (XW and YXZ) independently reviewed the EMRs. Finally, 111 patients were followed up for ambiguous medical histories or abnormal C4 levels, and 3 individuals were ultimately confirmed to have HAE.

### Identification of HAE by emrs: patient 1

A 64-year-old woman was admitted to the gastroenterology ward 6 years previously with recurrent abdominal pain. Her EMR documented intermittent abdominal pain during the previous 10 years, which had worsened during the last 3 years. The pain typically lasted 7–10 days, with a numeric rating scale (NRS) score of 8–9, and was relieved by defecation. There was no associated fever, vomiting, diarrhea, black stools, bloody stools, jaundice, rash, arthralgia, or changes in urine color. A prior ultrasound evaluation revealed approximately 400 mL of ascites in the abdominal cavity. The patient had undergone an appendectomy 30 years previously for acute appendicitis and was diagnosed with pancreatitis 20 years previously based on the presence of abdominal pain. During the hospitalization, she continued to experience abdominal pain. At that time, her C4 level was markedly low at 0.019 g/L (reference range: 0.21–0.39 g/L), anti-nuclear antibody (ANA) titer was elevated at 1:320 (cytoplasmic pattern: 1:160), and d-dimer level was increased to 1.44 mg/L FEU (reference range: 0–0.55 mg/L FEU). No abnormalities were detected in other examinations, including colonoscopy. The etiology of the abdominal pain remained unclear, but the pain resolved spontaneously, and the patient was subsequently discharged.

Considering her medical history and significantly decreased C4 level, a presumptive diagnosis of HAE was proposed. A follow-up telephone consultation was conducted to obtain additional medical history. The patient reported experiencing intermittent episodes of severe abdominal pain since the age of 10 years, with past diagnoses including biliary roundworms, kidney stones, appendicitis, and pancreatitis. Symptomatic treatment generally provided relief within 2 weeks. She also described episodic edema of the extremities since the age of 20 years, which typically resolved spontaneously within 7–8 days. There were no similar symptoms reported among her relatives. Subsequent evaluation included measurement of her C1-INH level and genetic screening, which revealed a C1-INH level of 0.25 g/L and a *SERPING1* (NM_000062.2): c.1396 C > T;p.(Arg466Cys) mutation. Although C1-INH function was not tested, the c.1396 C > T mutation has been reported to cause type 2 HAE; therefore, the patient was diagnosed with type 2 HAE. Long-term prophylactic treatment was not initiated because the patient had been free of edema and abdominal pain for the past 5 years. She was advised to prepare icatibant for potential future use, and regular follow-up was planned.

### Identification of HAE by emrs: patient 2

A 71-year-old woman was identified, and her EMR documented a history of episodic abdominal pain lasting more than 30 years with no clear etiology. She was followed up by telephone and called back for a comprehensive evaluation. Fifty-six years prior to the current presentation, she had experienced neck swelling without scratching, stinging, or a urticaria-like rash. Since then, she reported almost monthly episodes of self-limited swelling affecting the hands, feet, and extremities. These episodes were sometimes triggered by exposure to wind or menstruation but often occurred without an obvious cause, typically resolving within 1–3 days without treatment. Thirty-two years prior, she had experienced abdominal pain, nausea, and vomiting, which resolved within 1 day without specific treatment, but similar episodes recurred frequently over the subsequent 32 years. Twenty years prior, she had experienced facial edema followed by laryngeal edema, both resolving within 1–3 days without treatment. One week before her current evaluation, she developed edema of the face and extremities that subsided after 3 days without seeking medical attention. She reported no family history of edema, and her siblings and two children did not have similar symptoms. Laboratory results showed a C1-INH level of 0.04 g/L (reference range: 0.21–0.39 g/L), C1-INH function of 37.52% (reference range: ≥68%), and C4 level of 0.019 g/L (reference range: 0.100–0.400 g/L). Gene sequencing revealed the c.580G > T;p.(Glu194)* mutation in the *SERPING1* gene. She was diagnosed with HAE-1 but did not receive long-term prophylactic treatment because of the low frequency of her edema episodes. She was advised to use icatibant as needed, and regular follow-up was planned.

### Identification of HAE by emrs: patient 3

A 42-year-old woman presented with a 3-year history of episodic cutaneous edema and low C4 levels documented in her EMRs. She was subsequently followed up by telephone and recalled for laboratory testing. The patient reported recurrent edema of the extremities since the age of 39 years, with symptoms typically resolving within 3–5 days. She did not experience abdominal pain or laryngeal edema. Her son had similar symptoms but had not undergone C1-INH or C4 testing. Laboratory results revealed a C1-INH level of 0.09 g/L (reference range: 0.21–0.39 g/L) and a C4 level of 0.047 g/L (reference range: 0.100–0.400 g/L). The patient was ultimately diagnosed with HAE-1 and treated with icatibant during episodes of edema.

## Discussion

### Urgency of proactive diagnosis of rare diseases

“Rare” disease is a general term for a class of conditions with extremely low prevalence [3]. The relatively small number of patients with rare diseases highlights the difficulty of diagnosis and treatment. However, with as many as 10,000 rare diseases, it is not uncommon for people to suffer from these conditions [3]. Collectively, rare diseases are reported to affect 263–446 million people worldwide [4]. These diseases are often chronic, progressive, and life-threatening, and their diagnosis is hindered by a lack of awareness. Delayed diagnosis of rare diseases is a global challenge, with reports indicating it takes an average of 5 years and consultations with 7 physicians to achieve a diagnosis [18]. To address this, we propose refining the diagnostic process for rare diseases, as demonstrated by our study on HAE. Our goal is to alleviate the burdensome journey individuals often face, moving from one medical department to another in search of a diagnosis. By leveraging the proactive expertise of specialists and utilizing EMR databases, we can facilitate timely identification and diagnosis of rare diseases. This proactive approach not only promotes early diagnosis and treatment but also embodies the essence of medical service, ensuring prompt and accurate care.

The clinical symptoms of HAE include skin edema, abdominal pain caused by gastrointestinal edema, and dyspnea resulting from laryngeal edema. Gastrointestinal edema is often misdiagnosed as other common causes of abdominal pain. Our previous study revealed that 35.1%, 10.4%, and 6.5% of patients with HAE were misdiagnosed as gastroenteritis, appendicitis, and pancreatitis, respectively. Unnecessary surgeries, such as appendectomies or other laparotomies, were performed in 24.7% of these patients [19]. In addition, some individuals present with fatal laryngeal edema during their first attack, underscoring the critical need for early diagnosis [15]. In this study, three previously unrecognized cases of HAE were diagnosed. Two of these patients, whose diagnoses had been delayed for 54 and 56 years, had never visited the allergy department and were identified through a review of EMRs. The presence of potentially undiagnosed patients highlights an opportunity for clinicians to engage in data-driven, interdisciplinary collaborations to improve rare disease diagnosis in innovative ways.

### Role of EMRs in rare disease diagnosis

The reuse of EMRs and the development of disease networks provide new opportunities for diagnosing and managing diseases. Over the past decade, hospital adoption of EMRs has significantly increased [20]. Clinicians now spend more time interacting with computers, with this activity rising from 49 to 66% of their total time [21, 22]. EMRs store vast amounts of data that represent diverse clinical information, which can accelerate the discovery of new diseases, establish best practices for diagnosing complex cases, and support clinical decisions for challenging conditions. Several diagnostic and therapeutic advancements based on clinical data have already been reported. For example, the Undiagnosed Disease Network, launched by the NIH Clinical Center in 2008, has accelerated the diagnosis of rare or previously unrecognized diseases, improved their management, and contributed to medical science [23]. In 2011, Frankovich et al. [24] used EMRs at Stanford University School of Medicine to guide clinical decision-making for a young patient with systemic lupus erythematosus complicated by nephrotic-range proteinuria, antiphospholipid antibodies, and pancreatitis. At that time, there were neither guidelines nor publications to guide clinical decision-making. The clinicians queried similar cases in the EMR database and made decisions based on the best available data [24]. This example highlights how EMRs can enhance the practice of evidence-based medicine in the modern era.

### Limitations and future directions

We advocate for the proactive identification and diagnosis of individuals with rare diseases using EMRs or other clinical databases. This approach aims to identify undiagnosed patients, reduce diagnostic delays, and ensure timely and appropriate treatment. For hereditary diseases, identifying probands can help detect additional affected individuals within their lineage. To our knowledge, this is the first study to re-screen potential rare disease cases by reviewing their medical history in EMRs. However, some limitations should be acknowledged. First, in this study, specialists manually reviewed each case that met the screening criteria, which was time-consuming and labor-intensive. While machine learning-based computational models can potentially accelerate patient identification, these models face challenges, particularly in rare diseases, where the small number of confirmed cases for training results in inaccuracies [18]. Balancing human expertise with computational models is essential to facilitate early and proactive diagnosis. Second, our method relied on free-text search engines to identify patients with undiagnosed HAE by inputting terms describing symptoms. This strategy has limitations because physicians may use varying terms to describe the same symptom, leading to potential omissions despite our efforts to include all relevant terms. The use of Human Phenotype Ontology (HPO), a standardized vocabulary for phenotypic abnormalities, offers a promising solution [25]. HPO is widely used by researchers, clinicians, informaticians, and EMR systems worldwide and could enhance standardization and improve proactive diagnosis using either physician-led or artificial intelligence-based screening. Third, because of the high number of patients presenting with recurrent edema, abdominal pain, or laryngeal edema, we included a reduced C4 level as an additional inclusion criterion. This approach may have excluded patients with HAE who have normal C1-INH and C4 levels. Future clinical practice should address this limitation to ensure broader diagnostic coverage.

## Conclusions

This study introduced an innovative approach to the diagnostic process for rare diseases, aiming to reduce delays in diagnosis and treatment caused by individuals being referred between multiple hospitals or departments. Rare diseases often present with nonspecific and ambiguous symptoms, leading to diagnostic delays and suboptimal care. Our approach addresses this challenge by advocating for a more proactive role for physicians, leveraging their clinical expertise to identify potential cases of rare diseases at an earlier stage. By revisiting the EMRs of individuals with ambiguous symptoms, specialists actively identified and diagnosed rare diseases. Using HAE as an example, we confirmed 3 cases of HAE among 2,689 individuals presenting with symptoms such as skin edema, abdominal pain, or laryngeal edema, demonstrating the practicality of this proactive diagnostic model. Although the single-center nature of this study introduces geographical bias, limiting its generalizability to global screening for rare diseases, it presents a feasible approach and a conceptual shift in diagnostic practices. This methodology not only accelerates diagnosis and treatment but also significantly enhances individuals’ quality of life by reducing diagnostic delays.

## Data Availability

The datasets used and/or analyzed during the current study are available from the corresponding author on reasonable request.

## Declarations

### Confirmation of unpublished work

Our manuscript is original, has not been published before, is not currently being considered for publication elsewhere, and has not been posted to a preprint server.
